# Prevalence and predictors of unmet contraceptive need in HIV-positive female sex workers in Mombasa, Kenya

**DOI:** 10.1371/journal.pone.0218291

**Published:** 2019-06-19

**Authors:** Jessica E. Long, Gladys Waruguru, Krista Yuhas, Kate S. Wilson, Linnet N. Masese, George Wanje, John Kinuthia, Walter Jaoko, Kishorchandra N. Mandaliya, R. Scott McClelland

**Affiliations:** 1 Department of Epidemiology, University of Washington, Seattle, WA, United States of America; 2 Kenyatta National Hospital, Nairobi, Kenya; 3 Department of Global Health, University of Washington, Seattle, WA, United States of America; 4 Department of Medicine, University of Washington, Seattle, WA, United States of America; 5 University of Nairobi Institute of Tropical and Infectious Diseases (UNITID), University of Nairobi, Nairobi, GPO, Nairobi, Kenya; 6 Department of Medical Microbiology, University of Nairobi, GPO, Nairobi, Kenya; Sefako Makgatho Health Sciences University, SOUTH AFRICA

## Abstract

**Objectives:**

Female sex workers (FSWs) in sub-Saharan Africa are a key population for HIV prevention and treatment interventions, but less attention is given to their family planning needs. We evaluated the prevalence and predictors of unmet contraceptive need in HIV-positive FSWs.

**Study design:**

This cross-sectional analysis used data from an existing longitudinal study of FSWs in Mombasa, Kenya. This analysis included women who were HIV positive, age ≥18 years, pre-menopausal, not currently pregnant or desiring pregnancy, and reported exchanging sex for cash or in-kind payment at the time of enrollment. Unmet contraceptive need was defined as non-use of modern non-barrier contraceptives and not currently trying to become pregnant. Poisson regression was used to identify factors independently associated with unmet contraceptive need.

**Results:**

Among 346 HIV-positive FSWs, 125 (36.1%) reported modern non-barrier contraceptive use, leaving 221 (63.9%, 95%CI 58.8–68.9%) with unmet contraceptive need. Condom use was the only form of contraception for 129 (37.3%) participants. In unadjusted analyses, unmet contraceptive need was associated with physical abuse in the past year by someone other than a regular partner (PR 1.2, 95%CI 1.0–1.5), desire for (more) children (PR 1.3, 95%CI 1.1–1.5), and having 2–3 previous pregnancies compared to 0–1 prior pregnancies (PR 0.8, 95%CI 0.6–0.9). In adjusted analyses, lower number of previous pregnancies and having desire for future children remained significantly associated with a higher prevalence of unmet contraceptive need.

**Conclusions:**

Unmet need for modern non-barrier contraception was found in two-thirds of HIV-positive FSWs who reported that they were not currently trying to become pregnant, and was higher in women with the lowest number of prior pregnancies (0–1 prior pregnancies) and in those reporting desire for (more) children in the future. These findings highlight the need for concerted efforts to identify and eliminate barriers to contraceptive use in FSWs living with HIV.

## Introduction

In sub-Saharan Africa, 37% of female sex workers (FSWs) are estimated to be living with HIV, making this a key population for prevention and treatment interventions [1–3]. Considerable emphasis has been placed on HIV testing and condom promotion among FSWs to target the HIV epidemic [2,4]. In contrast, there has been little effort to address the family planning needs of FSWs [2,5,6]. Unintended pregnancy among FSWs can result in loss of clients, violence from partners, and financial burden [7]. Numerous studies in sub-Saharan Africa have reported high rates of unintended pregnancy and induced abortion, often illegal and conducted in unsafe conditions, as well as low levels of reliable contraceptive use in FSWs [6–12].

A systematic review of research conducted in Africa found limited coverage and a narrow scope of reproductive health services for this key population [2]. Many FSWs desire pregnancy prevention, but report significant barriers to contraceptive use including access to services, male partner preferences, cost, and misconceptions about side effects [13–15]. A qualitative study in Kenya found that FSWs reported unsupportive operational structure (e.g. long wait times, fees, and inconvenient hours) and discriminatory provider interactions as barriers to receiving reproductive health care [13]. These barriers contribute to unmet contraceptive need, defined as lack of contraceptive use in women of childbearing age who are not trying to become pregnant [16].

Few studies have examined the prevalence and predictors of unmet contraceptive need among African FSWs living with HIV [10,17]. In Kenya, an estimated 45% of FSWs are living with HIV [1]. Accurate information about unmet contraceptive need is vital to inform development of comprehensive HIV care and sexual and reproductive health packages for this population. Further, while condom promotion is common among FSWs, condom use alone may not offer adequate contraceptive protection in this population, as FSWs may not have agency to use condoms during all sex acts with client partners or replace condoms if they break [13,18]. This study evaluated the prevalence and predictors of unmet need for modern non-barrier contraception in FSWs living with HIV in Mombasa, Kenya.

## Methods

### Procedures

This cross-sectional analysis used data collected during enrollment into a prospective cohort study examining risk factors for HIV transmission from HIV-positive FSWs. Detailed methods have been published [19]. Briefly, eligibility criteria included laboratory confirmed HIV infection, age ≥18 years, and self-report of exchanging sex for cash or in-kind payment at the time of enrollment. In this analysis, women who were post-menopausal, trying to become pregnant, currently pregnant, or in the first six weeks of the postpartum period, were excluded. Recruitment was conducted through community outreach activities at bars, nightclubs, brothels, and other FSW workplaces and enrollment was conducted at the research clinic. All participants received free outpatient care at the clinic, including STI screening and treatment, and antiretroviral therapy (ART) if eligible according to Kenyan National Guidelines. All participants were reimbursed 250 Kenyan shillings (~$2.50 US dollars) for travel.

### Measures

At enrollment, a standardized face-to-face interview was conducted by a study nurse, collecting information on socio-demographics, health, and behavior. A physical examination was performed, including speculum-assisted pelvic examination with collection of genital swabs for STI testing. The primary outcome for this analysis was unmet contraceptive need, defined as not using a modern non-barrier contraceptive method and not currently trying to become pregnant. These items were assessed using standardized questions adapted from the Kenya Demographic and Health Survey (DHS) [16]. To assess fertility desire, women were asked, “Do you want any (more) children?” Those who answered ‘yes’, were then asked about current fertility intent, “Are you trying to get pregnant now?” Those who answered ‘yes’ regarding current fertility intent were excluded from analyses, so fertility desire only assessed those who had future, but not current, desire for children. Use of contraception was assessed as “Current method of contraception,” and included condoms, short- and long-acting reversible contraceptives, and tubal ligation. This exploratory analysis evaluated multiple potential correlates of unmet contraceptive need including sociodemographic characteristics, HIV disclosure, interpersonal factors (identifying a regular partner, partner attitude toward pregnancy) [20], controlling behavior (defined as responding yes to at least one of seven statements: he tries to keep you from seeing your friends; he tries to restrict contact with your family of birth; he insists on knowing where you are at all times; he ignores you and treats you indifferently; he gets angry if you speak with another man; he is often suspicious that you are unfaithful; he expects you to ask his permission before seeking health care for yourself), reproductive health factors (number of previous pregnancies, desire for children in the future), depression, alcohol use, and history of violence with both regular partners and others (e.g. clients) [21]. A regular partner was defined as a partner that was not a sex work client and was someone with whom the woman had an emotional relationship.

### Analysis

The prevalence of unmet contraceptive need was calculated as a crude proportion with 95% confidence intervals (CIs). Potential correlates of unmet contraceptive need were evaluated with unadjusted and adjusted Poisson regression with robust variance, which generated prevalence ratios (PR) and 95% CIs. Variables associated with unmet contraceptive need in unadjusted analyses (p<0.10) were retained in the adjusted model. In this exploratory analysis, an α level of 0.05 was used to define statistical significance all comparisons.

This study was approved by the ethics committees of Kenyatta National Hospital and the University of Washington. All participants provided written informed consent.

## Results

Between October 2012 and April 2017, 471 HIV-positive FSWs were enrolled. For this analysis, 125 participants were excluded (69 post-menopausal, 51 trying to become pregnant, 3 pregnant, 2 missing data on contraceptive use) leaving an analysis population of 346. Their median age was 38 years (interquartile range [IQR] 31–42), and 263 (76.0%) reported ever being married (Table 1). The median number of reported pregnancies was 3 (IQR 2–4) and 67 (19.4%) women reported a desire to have children (or have more children) in the future. Half of the women reported experiencing controlling behavior (172, 49.9%), and 74 (21.4%) reported intimate partner violence by their current or most recent regular partner in the past 12 months.

**Table 1:** Enrollment characteristics of 346 HIV-positive female sex workers.

Characteristic	Median (IQR) or n (%)
Age	38 (31, 42)
Highest education level ≥ 8 years	213 (61.6)
Ever married	263 (76.0)
Workplace	
Bar/restaurant	186 (53.8)
Nightclub	107 (30.9)
Home/other	53 (15.3)
Years since first sex work	
Less than 5	87 (25.1)
5 to 9	114 (33.0)
10 or more	145 (41.9)
Interpersonal factors	
Current or recent regular partner	286 (82.7)
No regular partner	60 (17.3)
Partner factors	
Regular partner in past 3 months^a^	170 (49.3)
Casual partner in past 3 months	180 (52.0)
Current or most recent regular partner attitude about pregnancy^b^	
Excited	150 (52.8)
Neutral	75 (26.4)
Upset	59 (20.8)
Contraceptive use	
None	92 (26.6)
Condoms only	129 (37.3)
Short / medium acting (DMPA, OCP)	79 (22.8)
Long acting (Implants, TL, IUCD, hysterectomy)	46 (13.3)
Reproductive health factors	
Number of previous pregnancies	3 (2, 4)
Wants to have children in the future	67 (19.4)
Self-reported sexual behavior in the past 7 days	
Condomless sex	30 (8.7)
Abstinent in past week	120 (34.7)
Subset of women not abstinent in past week^c^:	
i. 100% condom use in past week	196 (86.7)
ii. Number of sex acts in past week	2 (1, 4)
iii. Number of sex partners in past week	2 (1, 3)
iv. Any anal sex	1 (0.4)
Depressive symptoms by PHQ-9	
Minimal (0 to 4)	237 (68.5)
Mild (5 to 9)	73 (21.1)
Moderate / severe (10 or higher)	36 (10.4)
Alcohol use by AUDIT^a^	
Non-drinkers	152 (44.1)
Minimal (1 to 6)	103 (29.9)
Moderate (7 to 15)	72 (20.9)
Severe (16 or higher)	18 (5.2)
HIV disclosure	
Not disclosed to anyone outside healthcare setting	132 (38.2)
Disclosed to someone (e.g. acquaintances, religious leader, etc.) but not regular partner (husband, boyfriend/partner)	151 (43.6)
Disclosed to a regular partner	63 (18.2)
History of violence and controlling behaviors	
Sexual abuse in the past 12 months^d^^,^^e^	32 (9.3)
Physical abuse in the past 12 months^e^	31 (9.0)
Ever had controlling behaviors by a regular partner^a^^,^^f^	172 (49.9)
Any intimate partner violence in the past 12 months^f^	74 (21.4)

IQR: Interquartile range; AUDIT: Alcohol Use Disorders Identification Test [39]

^a^Data missing for 1 participant

^b^Included 284 women who reported that they had a current or most recent regular partner.

^c^Included 226 women who reported sexual behavior in the past 7 days

^d^Data missing for 2 participants

^e^By someone other than their current or most recent regular partner

^f^Intimate partner violence and controlling behavior are reported among their current or most recent regular partner. Regular partner was defined as a partner that was not a sex work client and was someone with whom the woman had an emotional relationship.

Modern non-barrier contraceptive use was reported by 125 (36.1%) women, leaving 221 (63.9%; 95%CI 58.8–68.9%) with unmet contraceptive need. Self-reported condom use was high, with 196 (86.7%) women reporting 100% condom use in the past week. For 129 (37.3%) women, condoms were the only form of contraception.

In unadjusted analysis, physical abuse in the past year by someone other than the current or most recent regular partner (PR 1.2, 95%CI 1.0–1.5) and future desire for more children (PR 1.3, 95%CI 1.1–1.5) were associated with significantly higher prevalence of unmet contraceptive need (Table 2). Compared to 0–1 pregnancies, having 2–3 prior pregnancies was negatively associated with unmet contraceptive need (PR 0.8, 95%CI 0.6–0.9). In adjusted analysis, desire to have children in the future (aPR 1.3 95%CI 1.1–1.5) and having fewer prior pregnancies remained significantly associated with unmet contraceptive need (aPR 0.8, 95%CI 0.7–1.0 in women with 2–3 prior pregnancies vs. 0–1 prior pregnancies).

**Table 2:** Unadjusted and adjusted association between baseline variables and unmet contraceptive need in HIV-positive Kenyan female sex workers.

Characteristic	N	n (%) with unmet contraceptive need	Unadjusted PR (95% CI)	p value	Adjusted PR (95% CI)	p value
Age, years				0.43		
20–29	72	41 (56.9)	1			
30–39	141	92 (65.3)	1.2 (0.9, 1.5)		—	
≥ 40	133	88 (66.2)	1.2 (0.9, 1.5)		—	
Highest education level				0.35		
< 8 years	133	89 (66.9)	1			
≥ 8 years	213	132 (62.0)	0.9 (0.8, 1.1)		—	
Marital status				0.45		
Never married	83	50 (60.2)	1			
Married, widowed, separated, or divorced	263	171 (65.0)	1.1 (0.9, 1.3)		—	
Workplace				0.02		0.11
Bar/restaurant	186	113 (60.8)	1		1	
Nightclub	107	79 (73.8)	1.2 (1.0, 1.4)		1.1 (1.0, 1.3)	
Home/other	53	29 (54.7)	0.9 (0.7, 1.2)		0.9 (0.7, 1.2)	
Years since first sex work				0.59		
<5	87	54 (62.1)	1			
5–9	114	77 (67.5)	1.1 (0.9, 1.3)		—	
≥ 10	145	90 (62.1)	1.0 (0.8, 1.2)		—	
Reports having a current or recent regular partner^a^				0.51		
No	60	36 (60.0)	1			
Yes	286	185 (64.7)	1.1 (0.9, 1.4)		—	
Reports controlling behaviors by current or most recent regular partner				0.53		
No	173	108 (62.4)	1			
Yes	172	113 (65.7)	1.1 (0.9, 1.2)		—	
Intimate partner violence in the past 12 months^b^				0.39		
No	272	177 (65.1)	1			
Yes	74	44 (59.5)	0.9 (0.7, 1.1)		—	
Any sexual abuse in the past 12 months^c^				0.11		
No	312	196 (62.8)	1			
Yes	32	24 (75.0)	1.2 (1.0, 1.5)		—	
Any physical abuse in the past 12 months^c^				0.04		0.08
No	314	196 (62.4)	1		1	
Yes	31	24 (77.4)	1.2 (1.0, 1.5)		1.2 (1.0, 1.5)	
Number of previous pregnancies				0.01		0.04
0–1	72	55 (76.4)	1		1	
2–3	166	97 (58.4)	0.8 (0.6, 0.9)		0.8 (0.7, 1.0)	
≥ 4	108	69 (63.9)	0.8 (0.7, 1.0)		0.9 (0.7, 1.1)	
Wants to have (more) children in the future				<0.01		0.01
No	279	169 (60.6)	1		1	
Yes	67	52 (77.6)	1.3 (1.1, 1.5)		1.3 (1.1, 1.5)	
Depressive symptoms by PHQ-9				0.40		
Minimal (0 to 4)	237	147 (62.0)	1			
Mild (5 to 9)	73	48 (65.8)	1.1 (0.9, 1.3)		—	
Moderate / severe (10 or higher)	36	26 (72.2)	1.2 (0.9, 1.5)		—	
Alcohol use by AUDIT				0.62		
Non-drinkers	152	101 (66.5)	1			
Minimal (1 to 6)	103	66 (64.1)	1.0 (0.8, 1.2)		—	
Moderate (7 to 15)	72	41 (56.9)	0.9 (0.7, 1.1)		—	
Severe (16 or higher)	18	12 (66.7)	1.0 (0.7, 1.4)		—	
HIV disclosure				0.47		
Not disclosed to anyone	132	79 (59.9)	1			
Disclosed to someone but not regular partner	151	101 (66.9)	1.1 (0.9, 1.3)		—	
Disclosed to regular partner	63	41 (65.1)	1.1 (0.9, 1.4)		—	
Current or most recent regular partner attitude about pregnancy				0.95		
Excited	150	96 (64.0)	1			
Neutral	75	49 (65.3)	1.0 (0.8, 1.3)		—	
Upset	59	39 (66.1)	1.0 (0.8, 1.3)		—	

^a^ Regular partner was defined as a partner that was not a sex work client and was someone with whom the woman had an emotional relationship.

^b^ Intimate partner violence was reported as violence by their current or most recent regular partner.

^c^ Any sexual violence and any physical violence were reported as violence by someone other than their current or most recent regular partner

PR: prevalence ratio; CI: confidence interval; AUDIT: Alcohol Use Disorders Identification Test; all p-values presented are for global tests.

## Discussion

In this population of FSWs living with HIV who reported that they were not currently trying to become pregnant, nearly 90% of women reported condom use during all acts of sexual intercourse in the past week. However, two thirds of women had unmet need for modern non-barrier contraception. A higher prevalence of unmet contraceptive need was independently associated with lower number of previous pregnancies and with desire for more children in the future.

Despite recognition of the importance of family planning among key populations living with HIV [6,22], few studies have examined contraceptive need in FSWs living with HIV. A cross-sectional study of FSWs in Swaziland found that 47% of women had unmet need for modern non-barrier contraceptives [10]. In the Swaziland study, as well as an analysis of data from FSWs in Burkina Faso and Togo, HIV status was not significantly associated with non-barrier contraceptive use, and HIV-positive respondents were no more likely to consistently use condoms than HIV-negative FSWs [10,22]. Preliminary results of an ongoing study of HIV-positive FSWs in Dar es Salaam, Tanzania, found that 31% of participants had unmet need for modern, non-barrier contraception [23]. A number of studies have examined contraceptive use in FSWs without specifically focusing on HIV-positive women. In populations of FSWs from Côte d’Ivoire, Ethiopia, Swaziland, and Madagascar, unmet need for modern non-barrier contraceptive use ranged from 24% to 54% [7–12].

In Kenya, unmet contraceptive need among FSWs was assessed in a 2008 study conducted in the Naivasha and Changamwe districts. This study included both HIV-positive and HIV-negative FSWs, and found that only 13.6% had unmet contraceptive need [8]. However, 39% of women in this study reported condom use as their only form of contraception, and only 41% reported condom use at last sex with an emotional partner. While condoms are essential for reducing FSWs’ risk of STIs including HIV, condom use alone is an unreliable contraceptive method, with a 10–15% annual failure rate with typical use [24]. Additionally, FSWs often report difficulty negotiating condom use with clients [8,13], and may use condoms less with intimate or steady partners [10,13]. In light of the limitations of condoms as a sole contraceptive method, a number of recent studies of FSWs, including the analysis presented in this paper, have sought to quantify unmet need for modern non-barrier contraceptives.

The present analysis found that sex workers who had experienced physical or sexual violence from someone other than their primary partner (e.g. clients) in the past year had a nearly 25% higher prevalence of unmet contraceptive need. This finding was no longer statistically significant at alpha = 0.05 in the multivariable analysis. Nonetheless, this finding suggests the need for more research to clarify the presence, magnitude, and mechanisms that may link physical abuse by clients or other non-regular sexual partners with unmet contraceptive need. Previous research has found that violence against FSWs is associated with reduced ability to negotiate condom use [6,21,25]. As a result, women experiencing violence may be at particularly high risk for unplanned pregnancy.

Despite our exclusion of women who reported currently trying to become pregnant, desire for children in the future was high in this population, and was associated with unmet contraceptive need. In the absence of safer conception services that include antiretroviral therapy and possibly pre-exposure prophylaxis for HIV-negative sex partners, pregnancy attempts in FSWs living with HIV could increase the risk for both transmission to sexual partners and mother-to-child transmission [5,26–29]. In research conducted in Burkina Faso and Togo, 20% of FSWs were trying to conceive, and only 25% of HIV-positive respondents trying to conceive were on ART [5]. One strategy to address both unmet contraceptive need and safer conception would be increasing access to integrated family planning and HIV services [6,30]. Integrated services have been effective in reducing unmet contraceptive need in general population HIV-positive women [31–33], and a similar strategy could be adapted in the context of specialized services for FSWs [2,6,13]. Integrated services could also provide a platform to address related issues such as partner violence and other barriers to contraceptive use [6,21,34], as well as contraceptive strategies for FSWs who do desire pregnancy and may face stigma in other family planning settings [30,35,36]. Currently, little guidance exists on integration of reproductive care for FSWs living with HIV [23,30,35]. To our knowledge, only a single study has evaluated an integrated HIV and family planning service delivery approach for this has population [37]. There was little evidence of change in contraceptive use with the integrated model. The authors note that the lack of change may be due to cultural barriers and misinformation about the side effects of short- and long term contraceptives, and may be more successful if these barriers are addressed [37].

This analysis had several strengths. The sample size of almost 350 women was essential for performing a multivariable analysis to explore which of several variables were independently associated with unmet contraceptive need. Standardized and validated tools were used to measure intimate partner violence, alcohol use, and depression [20,38,39]. In addition, the study assessed fertility desire and intent using questions that are employed by DHS [16], which will facilitate comparison to other literature.

This analysis also had a number of limitations. First, the cross-sectional design does not provide evidence of a temporal relationship between exposures and unmet contraceptive need. Second, this exploratory analysis included multiple exposures without adjusting for multiple comparisons. Further research should be conducted to assess the reproducibility of these relationships. Third, self-reported variables were subject to social desirability and recall biases. Strategies to minimize this bias included interviewer training and use of standardized data collection tools following written standard operating procedures. Fourth, the data collected from this cohort did not include information on contraceptive history or side effects from contraceptives, which may have influenced women’s contraceptive choices. Fifth, our use of a binary variable to assess fertility intent may have resulted in misclassification, as ambivalence in fertility intent has been documented in previous studies [40,41]. Finally, unmet contraceptive need is likely to be context-specific. This study is among the first to examine unmet contraceptive need in African FSWs living with HIV. However, these results may not be generalizable to all FSWs, as the women in this population are part of a long-term research cohort, and findings may be different in other settings in sub-Saharan Africa with differing access and knowledge of contraception options. Additional studies of HIV-positive FSWs in other settings will be helpful in identifying both variations and common themes.

In conclusion, a high prevalence of unmet need for modern non-barrier contraceptives was identified in this population of Kenyan FSWs living with HIV. Despite success in implementing HIV treatment and prevention services for FSWs, a critical gap remains in meeting their contraceptive needs. Interventions are needed to address barriers to contraceptive use in this population. One potential solution would be expanded access to integrated HIV treatment and sexual and reproductive health services that target the women with the greatest need. The impact of integrating these services for FSWs living with HIV has not been fully explored, and presents a promising area of research to improve care delivery to this population.
